# How Is Working Memory Training Likely to Influence Academic Performance? Current Evidence and Methodological Considerations

**DOI:** 10.3389/fpsyg.2017.00069

**Published:** 2017-02-07

**Authors:** Sissela Bergman Nutley, Stina Söderqvist

**Affiliations:** Pearson Clinical Assessment, Clinical ResearchStockholm, Sweden

**Keywords:** working memory training, reading, mathematics, working memory, learning, academic performance

## Abstract

Working memory (WM) is one of our core cognitive functions, allowing us to keep information in mind for shorter periods of time and then work with this information. It is the gateway that information has to pass in order to be processed consciously. A well-functioning WM is therefore crucial for a number of everyday activities including learning and academic performance (Gathercole et al., 2003; Bull et al., 2008), which is the focus of this review. Specifically, we will review the research investigating whether improving WM capacity using Cogmed WM training can lead to improvements on academic performance. Emphasis is given to reviewing the theoretical principles upon which such investigations rely, in particular the complex relation between WM and mathematical and reading abilities during development and how these are likely to be influenced by training. We suggest two possible routes in which training can influence academic performance, one through an effect on learning capacity which would thus be evident with time and education, and one through an immediate effect on performance on reading and mathematical tasks. Based on the theoretical complexity described we highlight some methodological issues that are important to take into consideration when designing and interpreting research on WM training and academic performance, but that are nonetheless often overlooked in the current research literature. Finally, we will provide some suggestions for future research for advancing the understanding of WM training and its potential role in supporting academic attainment.

## Introduction

At the center of all conscious information processing lies **working memory** *(WM)*—a fragile system responsible for processing and temporarily storing information. It is fragile because it can only keep track of a few pieces of information at the same time, and this information can fade away after just a short period of time (seconds) or be pushed out by distracting stimuli (Goldman-Rakic, 1996; McNab and Klingberg, 2008). WM capacity (WMC) predicts school performance years later (Gathercole et al., 2003; Bull et al., 2008; Alloway and Alloway, 2010; Geary, 2011). Furthermore, WMC in preschoolers has been shown to predict future risk of dropping out of high school (Fitzpatrick et al., 2015). Children with reading and math difficulties often exhibit WM deficits (Siegel and Ryan, 1989; Swanson and Jerman, 2006) and developmental studies of children with poor WMC have also reported findings to suggest that the effect is cumulative across development, resulting in greater decrements in learning as a child gets older (Alloway et al., 2009). This notion has instigated a quest for effective early interventions—all with the higher purpose of supporting the learning of these children.

KEY CONCEPT 1 **Working memory *(WM)***

The brain's processing system that allows us to mentally work with a limited amount of information in the now. It is fundamental to all advanced thinking and in order to learn facts or skills, that information must first pass through working memory—our mental workbench, before becoming more stable long-term representations.

This review will expand on the results from Söderqvist and Bergman Nutley (2015) showing improvements in reading and math performance two years following **Cogmed Working Memory Training (CWMT)**, put these results in a wider theoretical context and discuss these in relation to conflicting results from other studies. As cognitive training programs differ in both their content and implementation, consequently showing significantly different effects between intervention types (Melby-Lervåg et al., 2016), this review will focus on the most widely studied WM training program, CWMT™ (Klingberg et al., 2002).

KEY CONCEPT 2 **Cogmed Working Memory Training (CWMT)**

A computerized intervention entailing 12 different visuo-spatial and verbal WM span tasks that are presented in a rotating schedule and adapt to the capacity level of the trainee. Training is typically implemented during a period of 5–7 weeks, 30–40 min/day, 5 days/week, with weekly support from a certified coach that ensures compliance to the program.

The overarching question is whether an increased WMC following CWMT will transfer to increased school performance. A straightforward question perhaps, but what does it actually entail? Are we asking whether training affects the skills and content already learned, or whether training will help acquiring new skills and content in the learning to come? Earlier generations of “cognitive training” research struggled with similar questions and for instance Sidney Strauss described this complexity as follows (Strauss, 1972, p. 331):

“…the level of a child's structural development determines the concepts he will learn. That is, the intellectual structure sets the limits for what can be learned. In this sense learning is subordinate to development.”

In Söderqvist and Bergman Nutley (2015), we discussed two theoretical routes through which improved WMC could impact academic outcomes, the **learning route** and the **performance route**. These two theoretical routes are in no way exclusive from one another, but they are nonetheless important to distinguish when designing an intervention study as their interaction and effects are otherwise likely to be overlooked. Since WM training does not include actual teaching or practice of reading or mathematical skills, their development will depend largely on the education students are or have been receiving, along with the development of other cognitive functions required for skill proficiency.

KEY CONCEPT 3 **Learning route**

The mechanism by which WM training can influence academic performance through improving learning capacity. This can result from increased attention in class and from an increased capacity to digest new knowledge. Effects acting through this route would be evident in the long-term with outcome measures matching the curricular content.

KEY CONCEPT 4 **Performance route**

The mechanism by which WM training can influence academic performance through WM's direct involvement in academic tasks. Effects impacting this route would accompany the increased WM capacity and be evident on outcome measures of already learned skills on tasks tapping WM to its limits.

While the field of cognitive training is still young, some researchers have been tempted to summarize the current results in meta-analyses, reaching the conclusion that WM training does not transfer to academic outcomes (Melby-Lervåg and Hulme, 2013; Melby-Lervåg et al., 2016). Although meta-analyses can add value in assessing the current status of a field, the conclusions will only reflect the individual studies and the design choices therein. It is important to remember the theoretical assumptions in these studies and how they have been operationalized in order to understand what some of these, perhaps premature, conclusions are telling us. Since most studies that have assessed transfer effects to academic performance have focused on reading and mathematics we will here briefly summarize the literature of WM's role for the two.

## The role of WM for reading ability

The cognitive mechanisms underlying acquisition of reading ability have been studied extensively over the past decades (Daneman et al., 1980) and the complexity of its nature is well-demonstrated (Carretti et al., 2009; Kudo et al., 2015). Becoming reading proficient requires a delicate interplay between different cognitive abilities along with formal instruction and practice. For instance, one study of beginning readers (7-year olds) found that phonological awareness predicted reading accuracy, while phonological awareness and verbal WM predicted reading comprehension (Leather and Henry, 1994). As reading proficiency involves both cognitive abilities and skill acquisition, the strength of the relation between WM and reading is likely to vary over time depending on both cognitive maturity and skill development (Christopher et al., 2012). In one such study, the relations between reading comprehension and different cognitive components were assessed in a longitudinal design in grades 1–3 (Seigneuric and Ehrlich, 2005). The results indicated that WM became an increasingly important predictor of reading comprehension with age and it was not until grade 3 that WMC independently explained variance in reading comprehension after controlling for decoding and vocabulary. At earlier reading stages, the influence of WM could be explained by its shared variance with decoding and vocabulary skills. This main finding has also been reported in slightly older children, where verbal WM predicted reading comprehension after controlling for WM's shared variance with other verbal abilities (Cain et al., 2004; Kibby et al., 2014), however WM did not predict word identification (Kibby et al., 2014). Thus, WM is on an *independent* predictor of reading comprehension once word reading ability has been mastered.

Others have found that different aspects of WM seem related to different aspects of reading (Oakhill et al., 2011; Gathercole et al., 2016) and that this relation also varies between languages (Arina et al., 2015). The relation observable between WM and reading ability will thus depend partly on the types of assessments used to measure each construct. For example in the study by Seigneuric and Ehrlich (2005), correlation between WMC and reading in grade 3 was more prominent when using passage comprehension compared with a sentence comprehension outcome. Taken together, it seems that WM may be differentially supporting the two different phases of “learning to read” and “reading to learn” (Chall, 1996), in that WM may be transitioning from being one of several essential parts of the puzzle in reading acquisition to later becoming a crucial function in content understanding.

### How is WM training likely to influence reading?

As we have reviewed above, WM is only one of several capacities that are necessary for reading proficiency. Thus, the right question to ask may not be whether CWMT is likely to influence reading proficiency or not, but rather explore the stages where CWMT is likely to have effects on word decoding, reading fluency and reading comprehension respectively, depending on the baseline profile (cognitive and skill acquisition) of the child. For instance, phonological awareness has a more significant impact on reading acquisition in the early stages than WM. However, if WM is also impaired, then additional struggles are likely to cause hindrance in decoding progress, which could then be improved with CWMT. Conversely, if another ability than WM is acting as a bottleneck for a specific skill acquisition, then CWMT *alone* is not likely to have an effect on that skill. Since previous research has shown WMC to be predictive of certain aspects of reading at certain ages only, selecting appropriate outcome measures for training studies is crucial. Part of the variance seen between controlled published CWMT studies may indeed lay in the assessments chosen (see Table 1).

**Table 1:** **Effect sizes (Cohen's *d*) extracted from published studies on CWMT on developing samples reporting data on different aspects of reading**.

**Study**	**Sample**	**Age**	**Reading outcome**	**Reading category**	**Effect size**	**Follow up (>6 months) Effect size**
Dunning et al., 2013	Low WM, *n* = 94 Effect sizes in comparison with the active control group	7–9	Word reading (WORD)	Decoding	0.36	0.14^‡^
			Reading ability (NARA)	Passage comprehension	0.40	0.0^‡^
				Reading accuracy	0.03	−0.22^‡^
				Reading rate	0.04	−0.66^‡^
			Written expression (KTEA)	Written expression	0.69	
Holmes et al., 2009	Low WM, *n* = 42	8–11	Word reading (WORD/WIAT)	Decoding	0.01	0.07 (no control)
Dahlin, 2010	Special ed (mixed ADHD/ADD), *n* = 57	9–12	Reading comprehension (PIRLS)	Passage comprehension	0.88^*^	0.91^*^
			Word decoding/Non-words Orthographical test	Decoding	0.37	0.17
				Orthographical knowledge	−0.39	−0.13
Gray et al., 2012	Severe LD and ADHD and previously shown to be intervention resistant, *n* = 52	12–17	Sentence comprehension (WRAT)	Sentence comprehension	0.05	
Chacko et al., 2014	ADHD, high comorbidity (CD/ODD), *n* = 85	7–11	Sentence comprehension	Sentence comprehension	0.31	
			(WRAT)		−0.05	
			Word reading (WRAT)	Decoding	0.13	
			Spelling (WRAT)	Spelling		
Egeland et al., 2013	ADHD, *n* = 67	10–12	Decoding quality (LOGOS)	Decoding	0.57^*^	0.64^*^
			Decoding rate (LOGOS)	Speed of decoding	−0.32	−0.15
			Reading fluency & comprehension (LOGOS)	Passage fluency & comprehension	0.46^*^	0.62^*^
			Reading rate (LOGOS)	Reading speed	0.42^*^	0.17^*^
Foy and Mann, 2014	At risk academic progress, *n* = 50	4–6	First sound fluency test (DIBELSNext)	Phoneme awareness/Decoding		0.71 + (3 months)
Holmes and Gathercole, 2014	Low academic progress, *n* = 75 × 2	9–10	National standard	Composite of reading, writing, speaking and listening skills		−0.56
		10–11	assessment test (SAT) in English			0.67^*^
Partanen et al., 2015	Special education, *n* = 64	8–9	Diagnostic reading and	Passage comprehension	−0.15	0.07
			writing test (DLS, Swedish)	Phonological ability	−0.40	−0.69
				Word comprehension	−0.16	−0.05
				Spelling ability	−0.40	−0.14
Conklin et al., 2015	Cancer survivors, *n* = 62	8–16	Woodcock Johnson reading fluency	Sentence comprehension	−0.34	
Söderqvist and Bergman Nutley, 2015	Typically developing, *n* = 42	10–11	Diagnostic reading and writing test (DLS, Swedish)	Passage comprehension		0.66^*^ (24 months)
Bigorra et al., 2016	ADHD, *n* = 61	7–12	Pruebas psicopedagogicas de aprendizajes instrumentales en Catalan (Catalan test)	Passage comprehension	−0.13	−0.1
Fälth et al., 2015	Typically developing, *n* = 32	7	“Words and images” word decoding	Decoding	1.09	1.24 (2 months)
Roberts et al., 2016	Screened for low WM, *n* = 452	6–7	Sentence comprehension	Sentence comprehension		−0.12^#^ (12 months)
			Word reading (WRAT-4)	Decoding		−0.14^#^ (12 months) −0.14^#^ (24 months)
				Spelling		−0.12^#^ (12 months)
Phillips et al., 2016	TBI, *n* = 27	8–15	Reading comprehension	Passage comprehension	0.17^*^	0.16 (3 months)
			Word reading (WIAT-II)	Decoding	−0.056	0.17^*^ (3 months)

In studies not reporting effect sizes, it was calculated subtracting the change score for the control group from the intervention group divided by the pooled standard deviation.

* Indicates p < 0.05 and + indicates p < 0.1.

‡ Based on less than a third of the original sample,

# *values indicate standardized differences between groups in follow-up scores only since there were no baseline measures collected. KTEA, Kaufman Test of Educational Attainment; NARA, Neale Analysis of Reading Ability test; WORD, Wechsler Objective Reading Dimensions; DIBELS, Dynamic Indicators of Basic Early Literacy Skills (DIBELSNext); WRAT, Wide Range Achievement Test; DLS, Diagnostiskt Läs och Skrivtest; WIAT, Wechsler Individual Achievement Test*.

**Figure 1:**
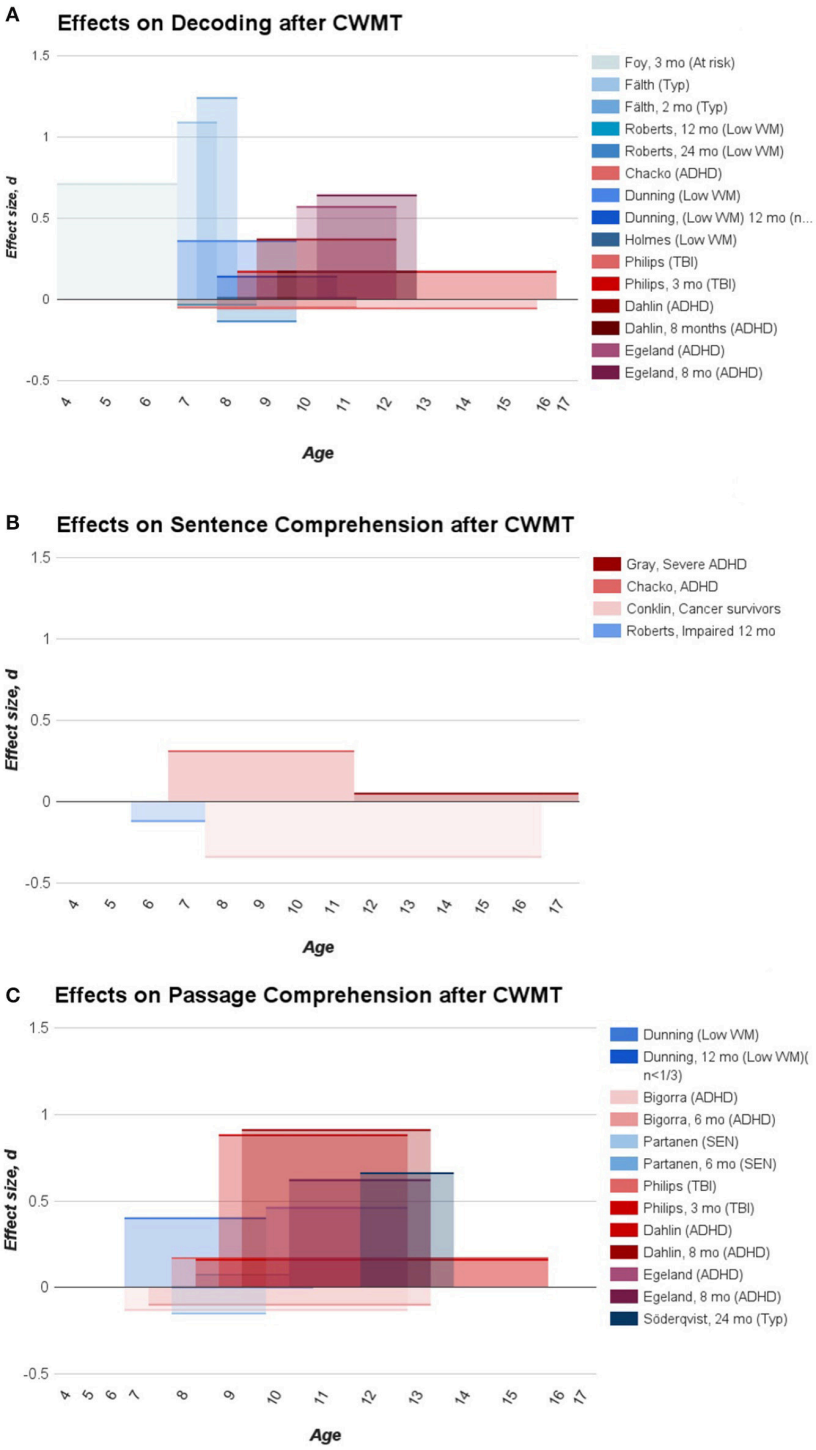
**Depicts the effect sizes (Cohen's *d*) extracted from the studies using CWMT divided between outcomes of decoding (A)**, sentence comprehension **(B)**, and passage comprehension **(C)**. Studies with clinical samples are color coded in a red shade whereas studies with samples without a clinical diagnosis are depicted in a blue shade.

For example, as WMC has not been shown to predict simple word recognition (e.g., Kibby et al., 2014) and reading comprehension when using simple sentences (compared to longer texts; Seigneuric and Ehrlich, 2005), these types of measures are not likely to be influenced by an improved WMC over and beyond a level that can be explained by variance common to verbal and phonological abilities. This is possibly reflected in a recent study (Roberts et al., 2016) that report no improvements in reading following CWMT in a large sample of 6 to 7-year-olds. The measures used to assess reading [from the Wide Range Achievement Test (WRAT)-4] at a 12 month follow up was word reading, sentence comprehension and spelling and at a 24 month follow up: word reading and spelling. Thus, considering the theoretical background discussed above it is not surprising that improvements were not observed on these measures. However, it would be inappropropriate to draw any strong conclusions regarding reading capacity in general as it remains unclear from this study whether effects would have been observed with other reading measures that are known to be more reliant on WMC (Dunning et al., 2013; Katz et al., 2016). When reading is assessed with passage comprehension on the other hand, it seems that CWMT more often than not has produced positive effects in both clinical and non-clinical samples, in line with what would be expected based on the literature reviewed above (Cain et al., 2004; Seigneuric and Ehrlich, 2005; Carretti et al., 2009; See Figure 1).

While the figure offer no clear explanation to the circumstances under which CWMT transfers to measures of reading, it highlights some of the complexities in interpreting the literature, namely the types of assessments used, the wide age range within studies, the learning status between studies and the time of the assessment. As previously discussed, WM seems to play a different role in supporting reading acquisition than it does reading comprehension, meaning that studies including students on both sides of becoming reading proficient (for instance in Phillips et al., 2016 ranging from age 8 to 16) are likely to see differential effects on the same outcome after training. It also seems that studies assessing reading proficiency in the early stages do tend to find effects on a group level on decoding or phoneme awareness (Foy and Mann, 2014; Fälth et al., 2015), whereas studies assessing older children on this measure tend to find effects primarily in impaired samples (Dahlin, 2010; Egeland et al., 2013), although not consistently (Chacko et al., 2014). This could perhaps reflect the different levels of restrainment WM has caused (on average) on the measured skill in the various study samples and to which degree other factors are hindering performance.

## The role of WM in mathematics

WMC predicts measures of current and future mathematical abilities (Passolunghi et al., 2007; De Smedt et al., 2009; Raghubar et al., 2010; Dumontheil and Klingberg, 2012; Peng et al., 2016) and their partly overlapping neuroanatomical correlates have been suggested to account for at least some of this observable relation (Zago et al., 2002; Swanson et al., 2008; Metcalfe et al., 2013). However, longitudinal studies have found different components of WM to be related to mathematics performance at different ages (De Smedt et al., 2009; Holmes et al., 2009; Raghubar et al., 2010) and between aspects of mathematics within the same age (Wiklund-Hörnqvist et al., 2016). The developmental stage of the participants does not only relate to the cognitive development (linked closely to age) but also to the quality and quantity of exposure participants have had to mathematical training (Morrison et al., 1997; Roberts et al., 2015). During early stages of learning arithmetic most children use counting strategies, at first often with the aid of fingers before developing verbal counting (De Smedt et al., 2009). Finally, the counting will gradually be replaced by forming categorical representations in long-term memory (LTM; Noël et al., 2004; De Smedt et al., 2009). The use of these different strategies put different demands on cognitive functioning, including that of WM. This is reflected in a changing pattern in how WM components relate to mathematics in that executive and visuo-spatial capacities appear to be mostly recruited for learning and application of new mathematical skills whereas the phonological loop/verbal WM becomes more important once the skill is learned (McKenzie et al., 2003; Raghubar et al., 2010). Thus, although the task is held constant between participants, the strategies used to solve it might differ and as a result associations with WM will differ between participants. Similarly, there might be intra-individual differences in cognitive demand if an individual is measured with a longitudinal design (or following an intervention) if the individual has switched strategy e.g., from verbal counting to automatized solutions between the measurement points. This is supported by a study by Meyer et al. (2010), who observed that while grade 3 students performed significantly better than grade 2 students on measures of operations and mathematical reasoning, no significant differences were observed in the WM measures found to correlate with these mathematical measures. This possibly reflects a development of strategies as a result of 1 year of formal education. Although these skills are reliant on WM, they are not necessarily reliant on a matched development of WM. Rather, a good fundamental WMC will allow for the learning and development of new skills and strategies (Bull et al., 2001) but once these are established WM will play a different role in performance (Imbo and Vandierendonck, 2007).

In addition, the term “mathematics” can include a large variety of skills. Geary (2011) describes two core domains: numerical facility (including skills such as arithmetic, number, and counting knowledge) and mathematical reasoning (representing more abstract mathematical knowledge). These two domains both rely on a number of cognitive functions, of which WM (and its different components) has been demonstrated to be one (Swanson and Jerman, 2006; Swanson et al., 2008; Geary, 2011). How WM relates to mathematics does not only depend on which domains of WM and mathematics are being assessed (Peng et al., 2016; Wiklund-Hörnqvist et al., 2016) but also on other aspects such developmental stage of the subjects and how tasks are presented (DeStefano et al., 2004).

### How is WM training likely to influence mathematical abilities?

As outlined above, WM is consistently found to relate to mathematical performance but the specific patterns of this relation are complex and not fully understood. Consequently, the effects of CWMT will be dependent on many different factors. For example, the effects from training are sensitive to the measures used, specifically as to which degree they tap WM. As we have discussed above, the load on these measures can differ across ages and specific strategies the child is using. The observation that the phonological loop seems to play a more important role for retrieving already learned skills in children (McKenzie et al., 2003; Raghubar et al., 2010) is important since capacity of the phonological loop is shown to be largely unaffected by CWMT (Holmes et al., 2009; Dunning et al., 2013). Thus, CWMT might have its potentially largest influence on the process of learning new skills or during more complex mathematical reasoning tasks shown to be more reliant on visuo-spatial resources (Holmes et al., 2006). These points have been largely overlooked in the majority of studies on CWMT to date and as can be seen from Table 2 and Figure 2, many studies have a wide age range of participants included. This will not only induce large variance due to development and strategies used, but in the cases where standardized assessments such as WRAT and Wechsler Individual Achievement Test (WIAT) have been used this will also mean that the actual tasks performed by the participants within the same study will differ due to the assessments wide inclusion of different mathematical domains (Raghubar et al., 2010) and the start and stop rules typically used within these assessments. These points make it difficult to interpret and generalize these results.

**Table 2:** **Effect sizes (Cohen's *d*) extracted from published studies on CWMT developing samples reporting data on different aspects of mathematics**.

**Study**	**Sample**	**Age**	**Math outcome**	**Math category**	**Effect Size**	**Follow up (>6 months) effect size**
Dunning et al., 2013	Low WM, *n* = 94 Effect sizes in comparison with the active control group	7–9	Maths reasoning (WOND)	Mix: problem solving, geometry etc.	−0.20	−0.27^‡^
			Number operation (WOND)	Written number operations	−0.4	
Holmes et al., 2009	Low WM, *n* = 42	8–11	Maths reasoning (WOND)	Mix: problem solving, geometry etc.	−0.08	(0.49 no control)
Dahlin, 2010	Special ed (mixed ADHD/ADD), *n* = 57	9–12	Basic number screening test	Number concepts and operations	0.69^*^	0.65
			Speeded additions verification tasks	Timed addition checking	0.55	0.33
			Speede subtraction verification tasks	Timed subtraction checking	0.01	−0.42
Chacko et al., 2014	ADHD, high comorbidity (CD/ODD), *n* = 85	7–11	Math computation (WRAT4)	Mix: number operations, counting, oral and written problems	0.1	
Egeland et al., 2013	ADHD, *n* = 67	10–12	Key math: math composite	Mix: a timed Mental computation subtest of verbally presented items and an untimed problem solving task with word problems	0.28	0.23
Bergman-Nutley and Klingberg, 2014	Low WM & ADHD, *n* = 480	7–15	Cogmed progress indicator	Multiple-choice timed arithmetic test	0.39^*^	
Holmes and Gathercole, 2014	Low academic progress, *n* = 75 × 2	9–10, 10–11	National standardized math tests	Mix: concepts and number operations, measuring, space, shapes and algebra		1.15^*^ 0.6^*^
Partanen et al., 2015	Special education, *n* = 64	8–9	Arithmetic (WISC-III)	Orally presented and solved number operations	−0.19	0.05
Conklin et al., 2015	Cancer survivors, *n* = 62	8–16	Woodcock Johnson math fluency	Timed written number operations	−0.13	
Söderqvist and Bergman Nutley, 2015	Typically developing, *n* = 42	10–11	Adler maths screener	Timed written number operations		0.58 + (24 months)
Phillips et al., 2016	TBI, *n* = 27	8–15	Numerical operations (WIAT-II)	Written number operations	−0.43	
Ang et al., 2015	Low WM, low math, *n* = 111 Effect sizes in comparison with the active control group	7	Numerical operations (WIAT)	Written number operations	−0.29	0.11
Roberts et al., 2016	Screened for low WM, *n* = 452	6–7	Math computation (WRAT-4)	Mix: Number operations, counting, oral and written problems		−0.18^#^ (12 months) −0.18^#^ (24 months)

In studies not reporting effect sizes, it was calculated subtracting the change score for the control group from the intervention group divided by the pooled standard deviation.

* Indicates p < 0.05 and + indicates p < 0.1.

‡ Based on less than a third of the original sample,

# *values indicate standardized differences between groups in follow-up scores only since there were no baseline measures collected. WOND, Wechsler Objective Number Dimensions; WRAT, Wide Range Achievement Test; WIAT, Wechsler Individual Achievement Test; WISC, Wechsler Intelligence Scale for Children*.

**Figure 2:**
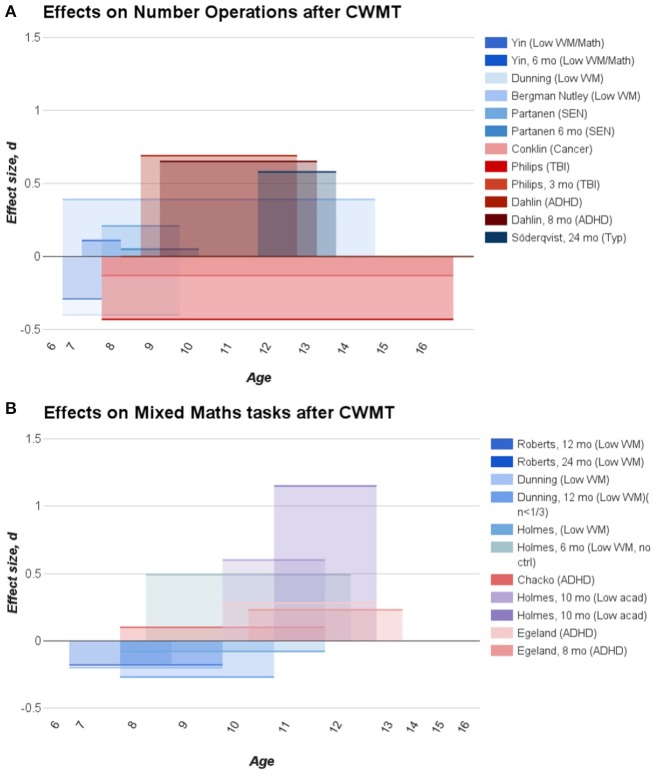
**Effect sizes (Cohen's *d*) extracted from published studies on CWMT reporting data on number operations (A)** and mixed maths tasks **(B)**. Studies with clinical samples are color coded in a red shade whereas studies with samples without a clinical diagnosis are depicted in a blue shade.

## Discussion

### Outcome measures to assess the performance route

As for all research, a crucial point to consider both when designing and interpreting research is whether the study design and outcome measures used actually answer the question(s) being asked. Most of the studies reviewed here set out to investigate whether effects from CWMT “transfers” or “generalizes” to academic performance (Gray et al., 2012; Egeland et al., 2013; Chacko et al., 2014; Foy and Mann, 2014) thus implicitly focusing on what has been discussed here as the *performance* route. This implies an assumption that WM would have been acting as a bottleneck for pre-existing skills and that increasing its capacity would unlock previously constrained academic potential. Within this line of reasoning, such effects would be apparent only on academic tasks with a WM load close to each subject's limits. Due to the complexity of both reading and mathematical learning it is also not obvious that improving WMC will lead to linearly associated improvements in the academic skills measured. One can imagine two alternatives, one in which a minimum level of capacity is required for simple mathematics or reading skills, such as for example identifying letters or reading and understanding a short and simple sentence. In this case, having a WMC above this threshold might not provide additional benefits on such tasks. On the other hand, more advanced reading tasks such as reading and understanding a whole paragraph of more complicated text might benefit from a higher WMC independently of the baseline, thus exhibiting a linear pattern of improvement with an increased WMC. Similarly to what Raghubar et al. (2010) have argued, the complexity of the relation between WMC and academic performance points to the need for task content analyses of outcome measures if we are to better understand when, how and for whom CWMT leads to significant transfer effects.

### Outcome measures to assess the learning route

Although learning itself takes time to manifest, there are other ways to study the process of learning as was done in a randomized, controlled study of children with ADHD (Green et al., 2012). After CWMT, children in the intervention group were observed to have fewer occurrences of looking away and playing with objects during an academic task compared with the children in the control group, concluding that CWMT had indirect impact on academic learning. Another study explicitly set out to assess both hypothetical routes of impact with assessments directly after training as well as after 12 months (Dunning et al., 2013). Other studies have investigated the *learning* route (Holmes and Gathercole, 2014; Söderqvist and Bergman Nutley, 2015; Roberts et al., 2016) only, that is, that CWMT would positively influence the learning capacity of the participants. This hypothesis can be simplified as:

CWMT + education > education alone

Within this premise, great care must be taken in selecting outcome measures that match the content of the education part of the equation. For example, while a well-functioning WM can help a child understand geometry, only specific instruction and practice will enable that child to solve a problem using pythagoras theorem (an example from the WIAT-II numerical operations subtask). Most of the studies discussed in this review have used short standardized assessments such as the WRAT and WIAT, and results from these have been used to generalize conclusions to the much wider term “academic achievement.” Although these are good measures for their own purpose, such as identifying individuals with specific learning difficulties, it is important to keep in mind that they only provide a snapshot of a student's academic abilities. It is therefore surprising that most studies using these have not included a discussion on how the particular tasks included (a) relate to WM and (b) for math primarily, match the curriculum to reflect what the students have been learning in school since the completion of training.

One approach that is more likely to capture progress on curricular content is to use metrics that schools already use, such as exams and national achievement measures, since these are specifically designed to capture learning progress. So far there have been two studies implementing CWMT in a school environment that have also used outcome measures based on assessments that the schools choose themselves as part of their typical academic assessment (Holmes and Gathercole, 2014; Söderqvist and Bergman Nutley, 2015). Both these studies stand out as finding significant improvements on mathematics and reading performance at long-term follow-up. It should however be noted that while year 6 students in the Holmes and Gathercole study demonstrated significant increases in both mathematics and English, the effects for year 5 students were less clear. Using established school metrics also has the benefit of the assessments being salient to the students since these contribute to grades and/or are presented in the usual educational context. Students might therefore be more motivated when performing these tests compared to tests performed for a research study only. Another potential benefit is reducing the risk of placebo effects driving the results. Although these studies have employed no-contact control conditions, placebo effects are unlikely to explain the results when using regular school based assessments administered by the teachers, about 10–24 months after the training, and with no obvious link to the study (Holmes and Gathercole, 2014; Söderqvist and Bergman Nutley, 2015).

### Time of assessment

Another requirement to assess the learning route is to allow for sufficient time between training and assessment for learning to take place. Although time increases the risk of introducing confounding factors, this should be avoided with well-controlled designs and should not hinder investigating effects on learning. This point is often disregarded in the literature. For example, a recent meta-analysis concluded that WM training (different programs bundled together) does not “generalize to important real-world cognitive skills, *even* when assessments take place immediately after training” (Melby-Lervåg et al., 2016). We believe that statements like these illustrate a lack of consideration for the different mechanisms underlying WM's role in learning, and thus how training is likely to influence academic performance.

### Training quality

Another overlooked factor when interpreting results from training studies is to consider how the training is implemented, not only with regards to compliance but also tracking the effort levels invested. Just as one would not expect to build muscles by going to the gym and simply sitting there, or lifting weights that are not putting a strain on the muscle, one should not expect effects from CWMT if a large portion of the training has been performed with low effort.

### Control conditions

A factor that has been more widely discussed is use of control groups (Morrison and Chein, 2011; Shipstead et al., 2012; Green et al., 2013). While active control groups are appropriate to control for test-retest and expectancy effects, their inclusion also warrants a close dissection of potential side-effects. For instance in a randomized, controlled trial studying 5–7 year old children with ADHD, the control condition consisted of the same tasks as the intervention group but with memory load set to 2 throughout the training (van Dongen-Boomsma et al., 2014). Baseline assessments showed that the sample had an average WMC of 2.6–2.8 indicating that the control group would have been training on a level that is likely to have trained their WMC. Consistent with this speculation, both groups showed improvements on many outcomes but the contrast between groups was scarce. Similarly, other samples of impaired individuals have used similar control conditions (Chacko et al., 2014) and even though the contrast may have been larger in older samples, it is still rather likely that training for 40 min/day on a low level task (similarly to a sustained attention task) could in fact lead to training effects, thus diluting the statistically measureable effects from the intervention. Such findings have been observed in brain activity after CWMT, showing similar changes in both groups, simply more pronounced in the intervention group (Brehmer et al., 2011). This emphasizes the importance of identifying the active ingredient one wishes to study, which will then guide the selection of an appropriate control condition.

If one wants to investigate whether CWMT + education > education, one should ensure that the education given to both groups is comparable for reliable conclusions to be drawn. This aspect might have influenced the results from the Roberts et al. (2016) study. In this study students were screened for WMC and those with low WMC were identified as at risk for academic underachievement. Half of these children were then informed of their deficits and selected to take part in a CWMT intervention. When doing so these children were taken out of class to perform the intervention, thus missing out on fundamental instruction. In this sense the Roberts et al. study rather investigated the hypothesis: CWMT > education. Improving the WMC of these students is unlikely to replace the education they have missed out on. Rather it runs the risk of them falling behind in their knowledge and skills, potentially resulting in negative influences on their self-esteem and motivation. Better design options to avoid this possibility is either performing the training outside of the school day as in Holmes and Gathercole (2014) or training whole classes and scheduling training to take time from several different subjects instead of one (as in Söderqvist and Bergman Nutley, 2015). A benefit of training whole classes is that all students miss the same content and the teachers can therefore compensate for this and no single student will suffer from falling behind in relation to their peers.

### Clinical vs. typically developing samples

The vast majority of studies reviewed herein have included children with some sort of cognitive deficit, such as low WMC, ADHD, or children receiving special education. Since these categories tend to include heterogeneous samples, often with high comorbidity with other deficits (Gray et al., 2012; Chacko et al., 2014) it is of particular importance to perform more in-depth analyses of the participants' characteristics and response to the intervention if we are to gain a deeper understanding of the results. A recent study including children with the age of 8–12 years and diagnosed with ADHD indicate that medication status and co-morbidity can both act as moderators for the effect following CWMT (van der Donk et al., 2016). Furthermore, baseline cognitive capacities have been found to be a predictor of both training improvements and transfer effects in a sample of children with intellectual disability (Söderqvist et al., 2012). As we have discussed above, if other deficits are present but not improved either by CWMT or by another parallel intervention, it is unlikely that any great improvements on transfer measures will be observed after CWMT alone. On the other hand, if WMC is the only or the most serious hindrance for performance, then CWMT is more likely to lead to noticeable improvements. However, such improvements run the risk of being missed in a classical group comparison design if there are other subgroups for which little or no improvements are observed. Performing in-depth analyses to understand inter-individual differences are necessary in order to understand when CWMT can lead to improvements in academic performance, and ultimately inform on how to create better individualized interventions.

In contrast, since our study (2015) included typically achieving children with no apparent deficits hindering their learning progress, it is more likely that effects from training will be more homogenous across the group. This study suggests that typically performing students can also benefit academically from CWMT.

### Power

An issue that most young intervention research fields suffer from is that of running studies with insufficient power to actually detect a true signal (Green et al., 2013). While small pilot studies can produce directionally informative results, their effect sizes will naturally rely on the heterogeneity of the sample and can thus be expected to vary between studies. Some have raised the issue of drawing invalid conclusions due to type-I errors (see for example Simons et al., 2016). The risk of type-II errors on the other hand is unfortunately seldom discussed in the cognitive training literature and as can be seen in Tables 1, 2 some studies report effect sizes in the range of 0.4–0.7 that are non-significant due to the small sample sizes in the studies (Dunning et al., 2013; Foy and Mann, 2014). However, these studies have concluded that “there were no effects” on these measures rather than stating that the results are inconclusive. This magnitude of effects has been deemed as relevant in educational interventions (Hattie, 2008) and highlights the importance of running studies with sufficient power to statistically detect such effects (as in for instance Bergman-Nutley and Klingberg, 2014; Roberts et al., 2016).

## Conclusion and future directions

In this review we have highlighted some important points to consider when designing future WM training studies, as well as when interpreting their results. As a whole, we believe the field would benefit from refocusing on the theoretical and functional underpinnings of the expected effects (e.g., Green et al., 2012), with design choices that reflect the complexity of the area. Based on the literature reviewed above, we consider WM's role in the *learning route* to be a promising notion to investigate further. In order to advance our understanding of how CWMT supports learning, we need to run larger studies that include more careful mapping of individuals' baseline profiles, and track long-term scholastic learning. Operationally, this would include consideration of how the intervention and outcome measures are matched with the education given to the participants along with in-depth analyses of inter-individual differences in responsiveness to training, and acknowledgement of how additional cognitive and educational skills interact with the outcome performance. This is particularly important for studies with clinical samples or academically underachieving children.

It is important to recognize that CWMT is not suggested, nor is it likely, to be a “magic pill” that solves all cognitive problems. Some of the effects are well-established whereas others are still in its early research days. Thus, there is still much to be learned about what an individual should expect from CWMT and grand scale studies are needed to answer the remaining questions outlined above. Caution should be given to not overstate effects, but just as importantly we should recognize that concluding at this early stage that WM training is ineffective without trying to understand the theoretical or functional mechanisms behind the effects, is premature. This runs the risk of leading to fewer intervention options for individuals who could benefit from them and cause stagnation of the research field and thereby our knowledge of training induced neuroplasticity. Let us instead move forward and look for solutions and deeper understanding of the mechanisms behind training and its effects.

## Author contribtions

All authors listed, have made substantial, direct, and intellectual contribution to the work, and approved it for publication.

### Conflict of interest statement

At time submission SN and SS are both employees of Pearson Clinical Assessment, the distributors of Cogmed Working Memory Training.
